# Dissecting causes for improved survival among patients with acute myeloid leukemia in two different eras receiving identical regimens in sequential randomized studies

**DOI:** 10.1038/s41408-018-0121-4

**Published:** 2018-08-22

**Authors:** Ing S. Tiong, John Reynolds, Kenneth F. Bradstock, John F. Seymour, Andrew H. Wei, on behalf of the Australasian Leukaemia & Lymphoma Group

**Affiliations:** 1 Department of Clinical Haematology, Alfred Hospital and Monash University, Melbourne, Australia; 2 Biostatistics Platform, Faculty of Medicine, Nursing and Health Sciences, Monash University, Melbourne, Australia; 3 Department of Haematology, Westmead Hospital and University of Sydney, Sydney, Australia; 4 Integrated Department of Haematology, Peter MacCallum Cancer Centre and Royal Melbourne Hospital, Melbourne, Australia

Cytarabine and anthracyclines represent the core therapeutic drugs for acute myeloid leukemia (AML). Despite the paucity of therapeutic innovations, large population-based registries have demonstrated incremental survival improvements over the decades, especially among younger patients^1,2^, potentially attributable to intensified chemotherapy, improved supportive care, or improved risk stratification and selection for allogeneic stem cell transplantation (SCT). However, it is difficult to ascertain the relative contribution of each to improvements in patient outcome.

The Australasian Leukemia and Lymphoma Group (ALLG) has conducted a series of randomized clinical trials in adult AML, leading to the stepwise incorporation of etoposide^3^ and high-dose cytarabine intensification in the induction phase^4^. Subsequently, both AMLM7 (1995–2000)^5^ and AMLM12 (2003–2013)^6^ trials, spanning an 18-year treatment period, used an identical induction protocol (ICE: idarubicin 9 mg/m^2^ days 1–3; cytarabine 3 g/m^2^ twice a day on days 1, 3, 5, 7; etoposide 75 mg/m^2^ days 1–7) and shared a common consolidation control arm (IcE: idarubicin days 1–2; cytarabine 100 mg/m^2^ days 1–5; etoposide days 1–5 × 2 cycles) as part of a 1:1 randomization with an investigational regimen in the post-remission phase. In AMLM7, the investigational arm included a second round of ICE, shown to be non-superior to standard IcE^5^. In AMLM12, anthracycline intensification incorporating an extra day of idarubicin in each of the two consolidation cycles was explored and shown to significantly improve leukemia-free survival^6^.

The overall survival (OS) in AMLM12 was superior to AMLM7 (median 44.3 vs. 24.8 months, *p* = 0.009) (Fig. 1a). To explore reasons for the differences in OS, we compared between each study era: (1) early induction outcomes following the identical ICE induction regimen; (2) post-remission outcomes in patients receiving the common standard IcE consolidation arm; and (3) survival following disease relapse.

**Fig. 1:**
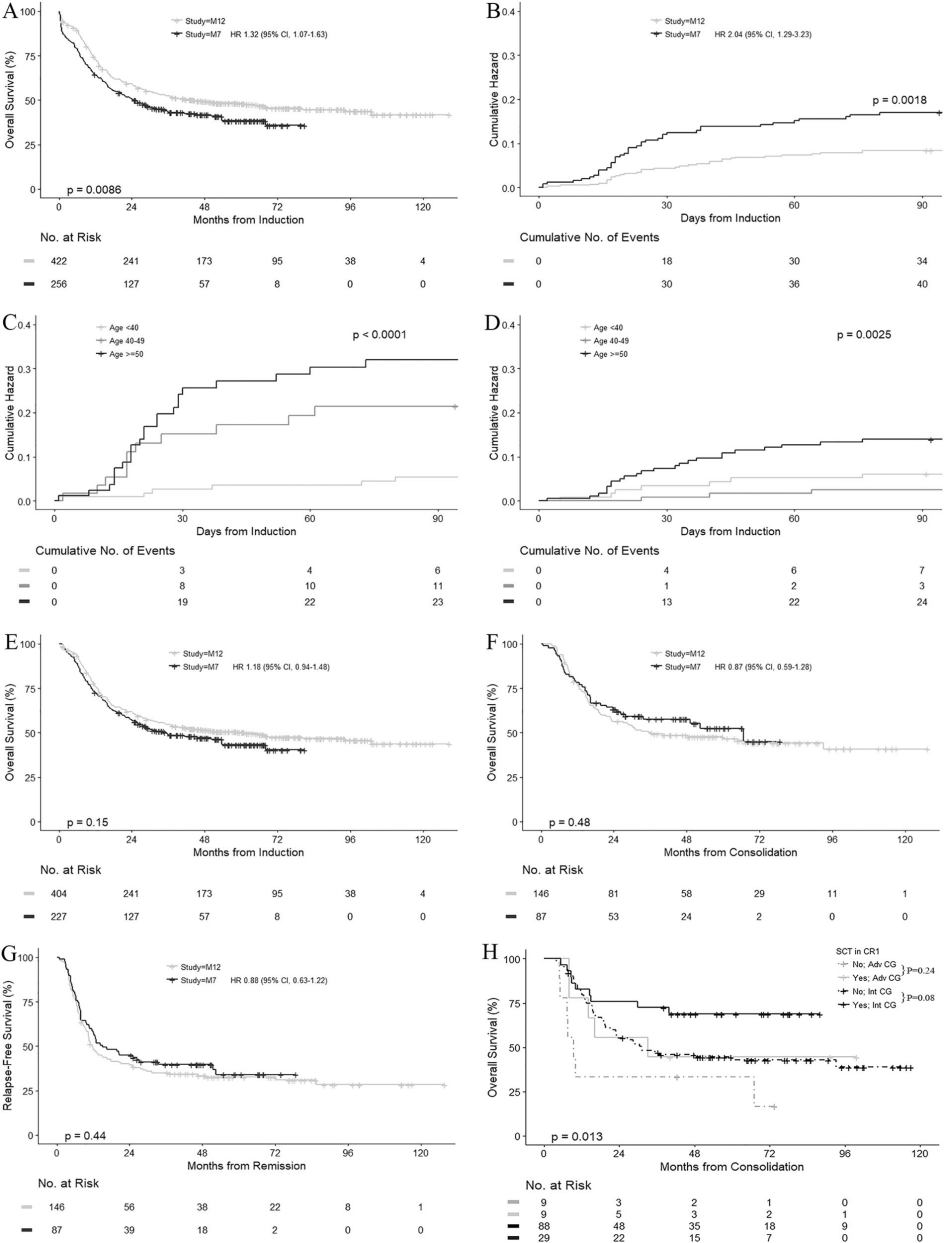
Overall survival, relapse-free survival and cumulative hazard curves. **a** Overall survival comparing the AMLM7 and AMLM12 study cohorts. **b** Cumulative hazard of early deaths in the AMLM7 and AMLM12 study cohorts. **c** Cumulative hazard of early deaths in the AMLM7 cohort stratified by age groups. **d** Cumulative hazard of early deaths in the AMLM12 cohort stratified by age groups. **e** Overall survival by landmark analysis at 30 days in the AMLM7 and AMLM12 study cohorts. **f** Overall survival of patients receiving the common IcE consolidation chemotherapy. **g** Relapse-free survival in patients receiving the common IcE consolidation chemotherapy. **h** Overall survival in the AMLM12 study cohort receiving standard IcE consolidation chemotherapy, restricted to patients with >90 days of remission duration, stratified by allogeneic SCT in first remission and cytogenetic risk. Pairwise comparisons using log-rank test, with *p*-values adjusted by Benamini & Hochberg (BH) method

Cytogenetic risk was classified in both studies in accordance with the revised MRC classification^7^. Patients with favorable-risk karyotype were excluded from the AMLM7 cohort as this subgroup was excluded in AMLM12. OS was calculated from the start of treatment and relapse-free survival from the date of first remission (CR1). Kaplan–Meier survival curves were compared using log-rank statistics, hazard ratios by Cox proportional hazard model, and univariate/multivariate analyses by logistic regression model. All tests were two-sided and considered significant where *p* < 0.05. R statistical software version 3.4.4 (R foundation for statistical computing, Vienna, Austria) was used.

Table 1 summarizes patient and treatment characteristics. The AMLM7 cohort was younger (median 43 vs. 48 years, *p* < 0.001), less commonly received granulocyte colony-stimulating factor (G-CSF), less commonly received allogeneic SCT and more commonly received autologous SCT. Complete remission (CR) rates (75% in AMLM7 and 76% in AMLM12) were similar after the first cycle of ICE. Median follow-up duration was 51.5 and 71.5 months for survivors in AMLM7 and AMLM12, respectively.

**Table 1:** Patient characteristics and outcomes in the entire AMLM7 and AMLM12 cohorts

	AMLM7 (*n* = 256)	AMLM12 (*n* = 422)	*p*-value^a^
Age, median years (IQR)	43.4 (31.6–51.6)	48.1 (38.4–55.2)	<0.001
Male gender (%)	55.1	57.1	0.6
ECOG PS > 0 (%)	50.0	54.9	0.2
*Cytogenetics, n (%)*			0.2
Intermediate	201 (78.5)	338 (80.1)	
Adverse	39 (15.2)	70 (16.6)	
Unknown	16 (6.3)	14 (3.3)	
*WCC (x 10* ^*9*^ */L), median (IQR)*	10.6 (3.3–43.5)	10.0 (2.9–33.7)	0.3
>40 × 10^9^/L (%)	26.2	21.6	0.2
>100 × 10^9^/L (%)	10.5	6.6	0.082
Febrile (%)	30.1	29.1	0.8
Bleeding (%)	22.3	12.8	0.002
DIC (%)	4.7	3.1	0.3
G-CSF (%)	80.1	96.7	<.001
>1 induction cycle, *n* (%)	19 (7.4)	41 (9.7)	0.3
CR—after 1 cycle (%)	75.4	76.3	0.8
CR—total (%)	79.7	82.5	0.4
*Randomized to standard IcE, n (%)*	87 (34.0)	146 (34.6)	0.9
Received 2 cycles of standard IcE	81 (93.1)	125 (85.6)	0.7
*Early mortality, n (%)*			
30-day mortality	30 (11.7)	18 (4.3)	<0.001
60-day mortality	36 (14.1)	30 (7.1)	0.005
90-day mortality	40 (15.6)	34 (8.1)	0.003
*Allogeneic SCT, n (%)*	76 (29.7)	215 (51.3)	<0.001
Transplant in first CR	32 (12.5)	120 (28.4)	<0.001
Transplant other status	44 (17.2)	95 (22.5)	0.12
Autologous SCT, *n* (%)	25 (9.8)	7 (1.7)	<0.001

^a^Fisher’s exact test for categorical variables; Mann-Whitney *U* tests for continuous variables

*CR* complete remission, *DIC* disseminated intravascular coagulation, *ECOG PS* Eastern Cooperative Oncology Group performance status, *G-CSF* granulocyte colony stimulating factor, *IQR* interquartile range, *SCT* stem cell transplantation, *WCC* white cell counts

We initially focused on early induction outcomes among 678 patients receiving the ICE induction protocol (*n* = 256 in AMLM7 and *n* = 422 in AMLM12). Early deaths (at 30 days) were more common in AMLM7 (11.7%) than AMLM12 (4.3%) (*p* = < 0.001) (Fig. 1b), and strongly linked to increasing age: with higher mortality occurring in patients >40 years in AMLM7 (Fig. 1c) but only >50 years in AMLM12 (Fig. 1d). Although OS appeared improved in the AMLM12 study (Supplementary Figure S1A), this difference in OS was attenuated if OS was compared using a 30-day landmark analysis (Fig. 1e), confirming the initial divergence of the survival curves seen in Fig. 1a. Higher 30-day mortality (odds ratio 3.46, 95% confidence interval, 1.83–6.69) was observed in the AMLM7 vs. the AMLM12 study eras (Supplementary Table S1). In addition to these inter-study differences, there also appeared a trend for reduced intra-study early deaths in the later half of the AMLM12 recruiting period (2007–2010), compared to the first half (2003–2006) (Supplementary Figure S1A-B).

The ICE induction protocol was identical except for the first 44 patients in the AMLM7 study who received idarubicin 12 mg/m^2^. Thirty-day mortality was not higher in this initial subgroup (4/44; 9.1%). The remaining patients in AMLM7 and all patients in AMLM12 received the identical induction and consolidation regimen in the control arm. Hence, the reduction in early deaths in AMLM12 was not related to differences in chemotherapy intensity, and was likely attributable to improved supportive care practices in the AMLM12 study. Aspects of supportive care were explored where data were available (Supplementary Table S2).

Antifungal prophylaxis was documented in 75/87 (86%) patients in AMLM7, all using fluconazole/itraconazole, and in 397/414 (96%) patients in AMLM12, using fluconazole/itraconazole (71%) or posaconazole/voriconazole (38%). Many patients received >1 antifungal agent at different time points. AMLM12 patients who received mold-active antifungal prophylaxis had a trend for reduced incidence of documented fungal infections (4.7 vs. 10.2%, *p* = 0.06), similar 30-day mortality (3.4 vs. 3.8%) and potentially lower 90-day mortality (4.0 vs. 9.1%, *p* = 0.07), consistent with previously demonstrated benefits of posaconazole over fluconazole or itraconazole^8^. Strict definitions of possible/probable/definite invasive fungal infections were not available in the database and so could not be compared. The availability of mold-active antifungal agent in the later era may have contributed to the improved outcomes observed for patients recruited to the second half of the AMLM12 study era (Supplementary Figure S1C).

Although the use of G-CSF (80% in AMLM7, 97% in AMLM12) and palifermin (0 vs. 18%) were more frequent in the later AMLM12 cohort, published randomized trials did not demonstrate significant impact of either agent on induction deaths^9,10^. Around one-third of patients received prophylactic fluoroquinolones in both cohorts, with similar fever days and documented infections. Transfusion support was also similar for both red blood cells and platelets. The leading causes of death for both cohorts, where documented, were infection (59% in AMLM7 and 39% in AMLM12) and multiorgan failure (28% in AMLM7 and 50% in M12).

After examining early induction outcomes, we next compared post-remission outcomes in 233 patients (*n* = 87 in AMLM7 and *n* = 146 in AMLM12) randomized to the IcE consolidation control arm common to both studies. Despite older age and more patients with ECOG > 0 in the AMLM12 cohort (Supplementary Table S3), there was no significant difference in either OS (median 36.4 months in AMLM12 vs. 66.7 months in AMLM7, *p* = 0.48) (Fig. 1f) or relapse-free survival (median 11.8 months vs. 15.1 months, *p* = 0.44) (Fig. 1g). Although significantly more patients underwent CR1 allogeneic SCT in AMLM12 (28 vs. 10%), long-term post-remission survival outcomes were identical, despite matching for baseline characteristics, censoring for SCT, or treating SCT as a competing risk (Supplementary Figures S2–S3). Allogeneic SCT in CR1, however, was associated with improved survival in patients with intermediate (*n* = 117), but not adverse cytogenetic risk (*n* = 18) in the AMLM12 cohort (Fig. 1h). In AMLM7, SCT frequency was too low to enable meaningful interpretation of its impact (Supplementary Figure S4).

Among the entire AMLM7 and AMLM12 cohorts, 114 patients and 207 patients, respectively, experienced disease relapse associated with limited OS (median 6.5 months vs. 7.9 months) (Supplementary Figure S5A). Data on salvage therapy and outcome were not available, but more patients in AMLM12 (47%) than AMLM7 (38%) underwent subsequent allogeneic SCT (*p* = 0.10), where survival was significantly improved, compared to patients not transplanted (Supplementary Figure S5B).

The goal of high-dose cytarabine-based induction is the rapid achievement of high-quality CR from the initial chemotherapy and reduce the likelihood of re-induction therapy, sparing patients the risk of additional complications. ICE results in a very high first-cycle CR rate (~76% excluding favorable-risk AML), compared to 59 and 71% after 1 and 2 cycles of 7 + 3 (90 mg/m^2^ daunorubicin)^11^. Our analysis of randomized clinical trials from two different eras demonstrates that ICE induction has become more tolerable over time, especially in those <50 years, likely from multi-faceted improvements in supportive care. Interestingly, a volume effect was evident in the AMLM12 (but not in the AMLM7 study), where patients in the top five recruiting centers had better survival outcome (Supplementary Figure S6); similar effects have been observed by others^12^. Early deaths from ICE induction in AMLM12 were 2.1 and 7.0% in patients <50 and ≥50 years. This compares favorably to other AML studies conducted in a similar era: 5.5% induction deaths in an ECOG study (2002–2008)^11^, and 5.5 and 10.1% in patients <46 and ≥46 years in the EORTC-GIMEMA AML-12 study (1999–2008)^13^.

Although a crude comparison between the AMLM12 and AMLM7 studies indicated a significant improvement in OS, potentially attributable to chemotherapy intensification in CR1, our analyses demonstrate that a substantial component of this benefit may also be linked to non-chemotherapy related factors. By examining deaths occurring during the induction and consolidation phases separately, we find that the major effect on survival was related to reduced treatment-related mortality during the induction phase of AMLM12 associated with improvements in supportive care practices, although the precise factors could not be fully determined. In the post-remission setting, there was no major difference in relapse-free or OS, despite the increased incidence of allogeneic SCT in the more recent AMLM12 study. In conclusion, these findings demonstrate the complexities in making interpretations between identical treatment regimens delivered in sequential eras and highlights the importance of ensuring major practice changing decisions are based on prospectively conducted randomized controlled trials.

## Electronic supplementary material

Supplementary Material
