# Supplementary material for: Dissecting causes for improved survival among patients with acute myeloid leukemia in two different eras receiving identical regimens in sequential randomized studies
Source: Blood Cancer J. 2018 Aug 22;8(9):84. doi: 10.1038/s41408-018-0121-4 (PMC6127140; doi:10.1038/s41408-018-0121-4)

Supplementary Appendix

This appendix has been provided by the authors to give readers additional information about their work.

Supplement to: Tiong IS, Reynolds J, Bradstock KF, Seymour JF, and Wei AH, on behalf of the Australasian Leukaemia & Lymphoma Group. Dissecting causes for improved survival among patients with acute myeloid leukemia in two different eras receiving identical regimens in sequential randomized studies.

Supplementary Tables: 3

Supplementary Figures: 6

Table S1. Univariate and multivariate analysis of predictor of 30-day mortality by logistic regression in the AMLM7 and AMLM12 study cohorts.

Factors	Univariate Analysis		Multivariate Analysis*	
	**Odds Ratio (95% CI)**	**P-value**	**Odds Ratio (95% CI)**	**P-value**
Cohort: AMLM7	2.85 (1.56–5.34)	.002	3.46 (1.83–6.69)	< .001
Age	1.07 (1.03–1.11)	< .001	1.08 (1.04–1.12)	< .001
Gender: Male	0.73 (0.40–1.32)	.3	-	
ECOG PS > 0	2.20 (1.18–4.31)	.017	1.76 (0.91–3.55)	.1
Adverse cytogenetics	1.38 (0.63–2.77)	.4	-	
WCC, every $10x10^9$/L	1.07 (1.02-1.11)	.003	1.06 (1.01–1.10)	.019
Febrile	1.39 (0.73–2.56)	.3	-	
Bleeding	1.62 (0.82–2.99)	.1	-	
DIC	2.69 (0.76–7.47)	.081	2.44 (0.63–7.74)	.2
G-CSF	0.58 (0.26–1.47)	.2	-	

***** Factors were included in the multivariate analysis if p<0.10 by univariate analysis.

Abbreviations: CI, confidence interval; DIC, disseminated intravascular coagulation; ECOG, Eastern Cooperative Oncology Group; G-CSF, granulocyte colony stimulating factor; PS, performance status; WCC, white cell counts.

Table S2. Supportive care in the AMLM7 and AMLM12 study cohorts.

	AMLM7	AMLM12	P-value
Prophylactic antifungal*, n (%)	75 (86.2)	397 (95.9)	.001
Fluconazole / Itraconazole	75 (100)	283 (71.3)	
Posaconazole / Voriconazole	-	149 (37.5)	
Amphotericin	3 (4.0)	33 (8.3)	
Infection, n (%)			.7
None	8 (8.3)	51 (12.0)	
Probable	21 (21.9)	84 (19.8)	
Clinical	11 (11.5)	43 (10.1)	
Microbiological	56 (58.3)	246 (58.0)	
Days febrile, median (IQR)	10.5 (5–17)	10 (6–15)	.7
G-CSF (%)	80.1	96.7	< .001
Palifermin, n (%)	-	74 (17.5)	
RBC (units), median (IQR)	10 (8–14)	12 (8–15)	.089
PLT (units), median (IQR)	9 (7–15)	10 (8–14)	.185

*Many patients received >1 antifungal agent at different time points.

Abbreviations: G-CSF, granulocyte colony stimulating factor; IQR, interquartile range; PLT, platelets; RBC, red blood cells.

Table S3. Characteristics of patients who received standard IcE consolidation from the AMLM7 and AMLM12 (original and matched) study cohorts.

	**AMLM7 (n=87)**	**M12_original (n=146)**	**P-value[a]**	**M12_matched[b] (n=87)**	**P-value[a]**
Age, median years (IQR)	41.8 (33.3–50.6)	48.0 (38.8–55.3)	.001	41.8 (33.2–52.7)	.6
Male gender (%)	52.9	53.4	1	51.7	1
ECOG PS > 0 (%)	44.8	59.4	.041	46.0	1
Cytogenetics, n (%)			.6		1
Intermediate	69 (79.3)	122 (83.6)		69 (79.3)	
Adverse	13 (14.9)	19 (13.0)		13 (14.9)	
Unknown	5 (5.8)	5 (3.4)		5 (5.8)	
WCC (x 10^9/L), median (IQR)	10.1 (3.9–31.0)	11.6 (3.9–37.5)	.6	11.3 (4.9–37.0)	.5
> 40 x 10^9/L (%)	16.1	24.0	.2	23.0	.3
> 100 x 10^9/L (%)	4.6	6.2	.8	3.5	1
>1 induction cycle, n (%)	3 (3.5)	9 (6.2)	.5	5 (5.7)	.7
Allo-SCT, n (%)	30 (34.5)	85 (58.2)	<.001	53 (60.9)	<.001
Transplant in first CR	9 (10.3)	39 (26.7)	.003	24 (27.6)	.006
Transplant other status	21 (24.1)	46 (31.5)	.3	29 (33.3)	.2
Auto-SCT, n (%)	12 (13.8)	4 (2.7)	.002	1 (1.2)	.002

[a] Fisher's exact test for categorical variables; Mann-Whitney *U* tests for continuous variables.
[b] Propensity score matching by regression including age, gender, WBC and cytogenetic risk group. Matching was done using the nearest neighbor method with 1:1 ratio without replacement.

Abbreviations: CR, complete remission; DIC, disseminated intravascular coagulation; ECOG PS, Eastern Cooperative Oncology Group performance status; G-CSF, granulocyte colony stimulating factor; IQR, interquartile range; SCT, stem cell transplantation; WCC, white cell counts.

Figure S1. (A) Overall survival and **(B)** cumulative hazard of early deaths in the entire AMLM7 and AMLM12 cohorts, stratified by period of study recruitment. **(C)** Percentage of patients (%) receiving GCSF (line and dots) and prophylactic posaconazole/voriconazole (bar chart) over time (AMLM7: 1995-2000; AMLM12: 2003-2010).

A

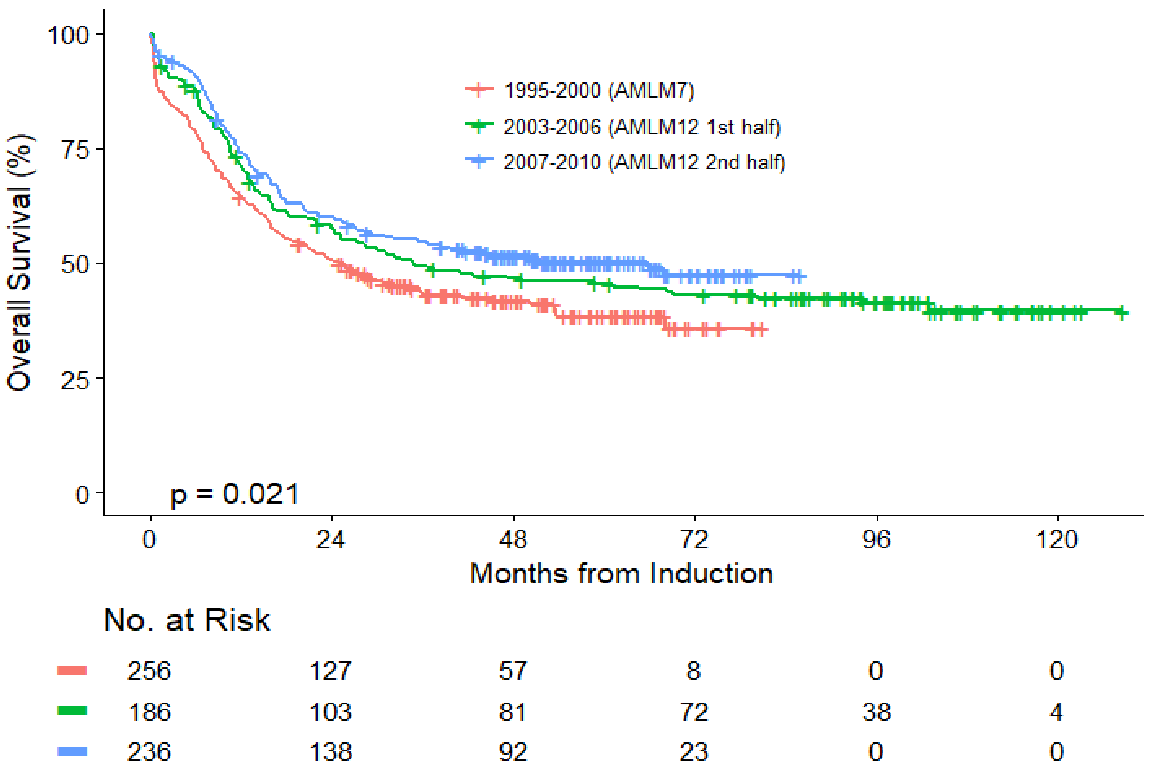

B

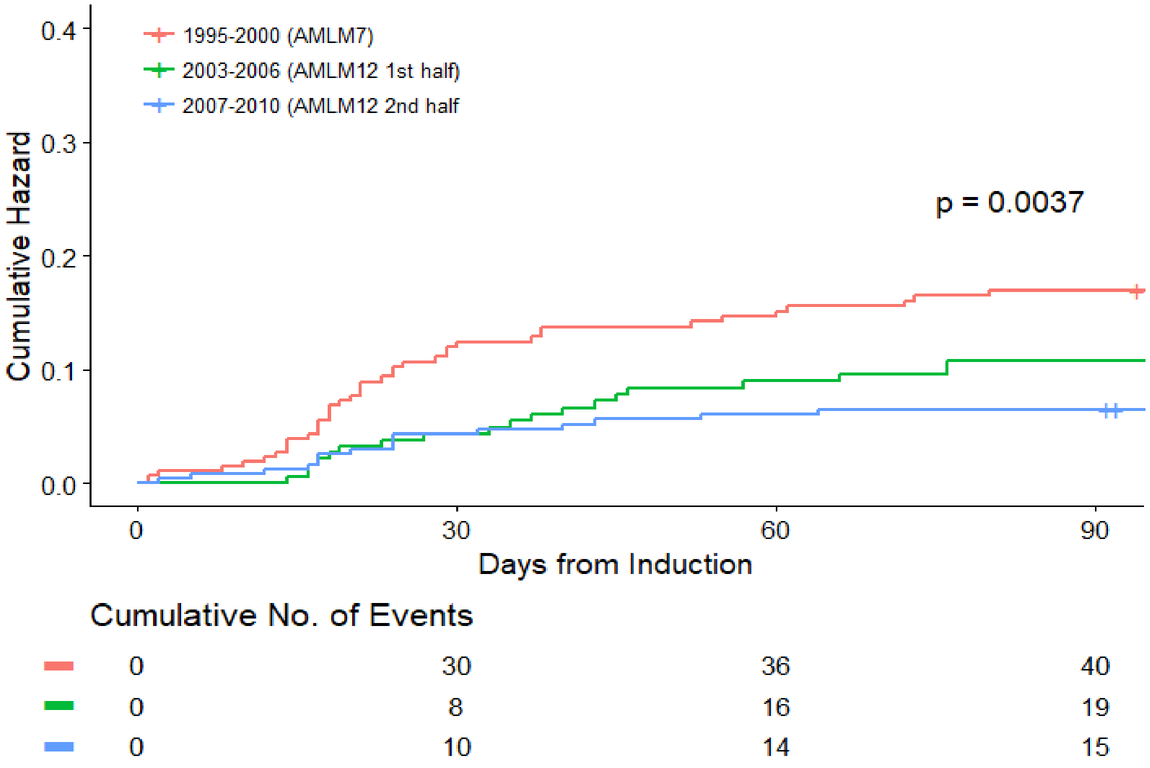

C

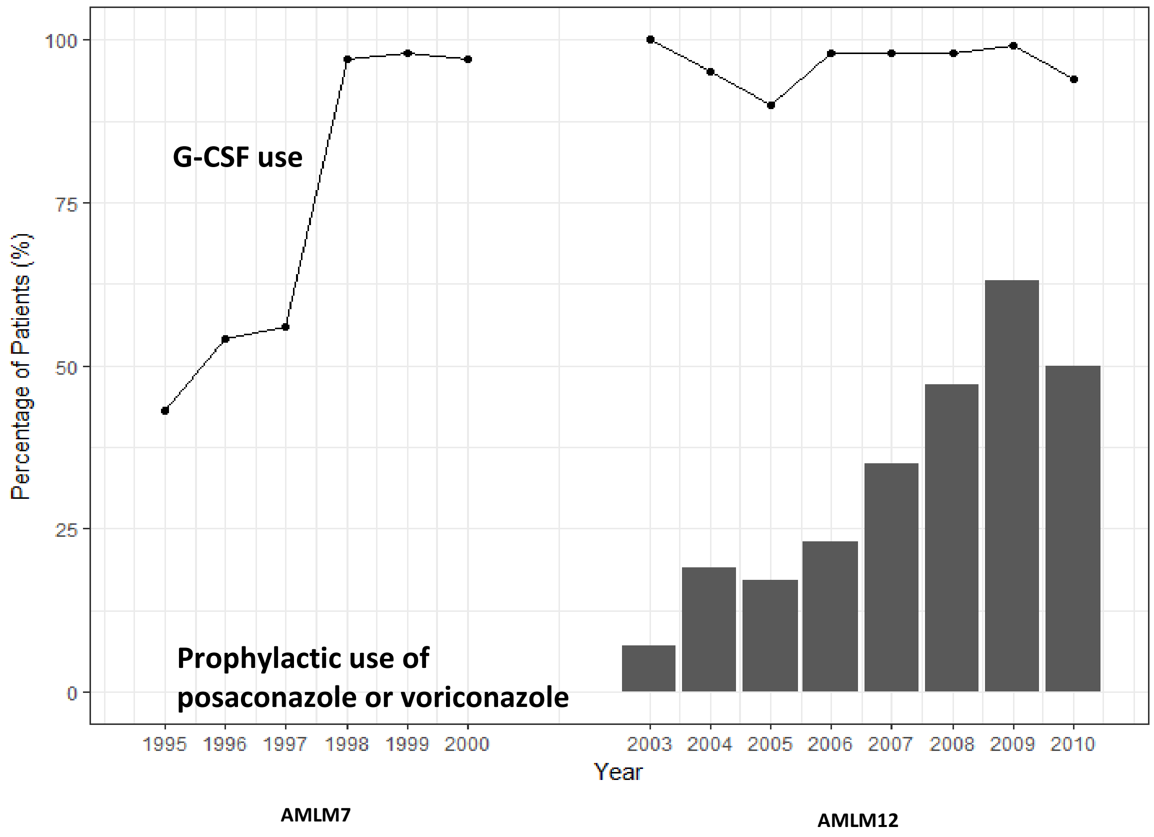
G-CSF use
Prophylactic use of
posaconazole or voriconazole
Percentage of Patients (%)
100
75
50
25
0
1995
1996
1997
1998
1999
2000
2003
2004
2005
2006
2007
2008
2009
2010
Year
AMLM7
AMLM12

Figure S2. Overall survival censoring for SCT (both autologous and allogeneic) in patients receiving standard IcE consolidation in the AMLM7 and AMLM12 study cohorts. **(A)** Unmatched comparison between study cohorts. **(B)** Propensity score matching for age, gender, ECOG, WCC and cytogenetic risk.

A

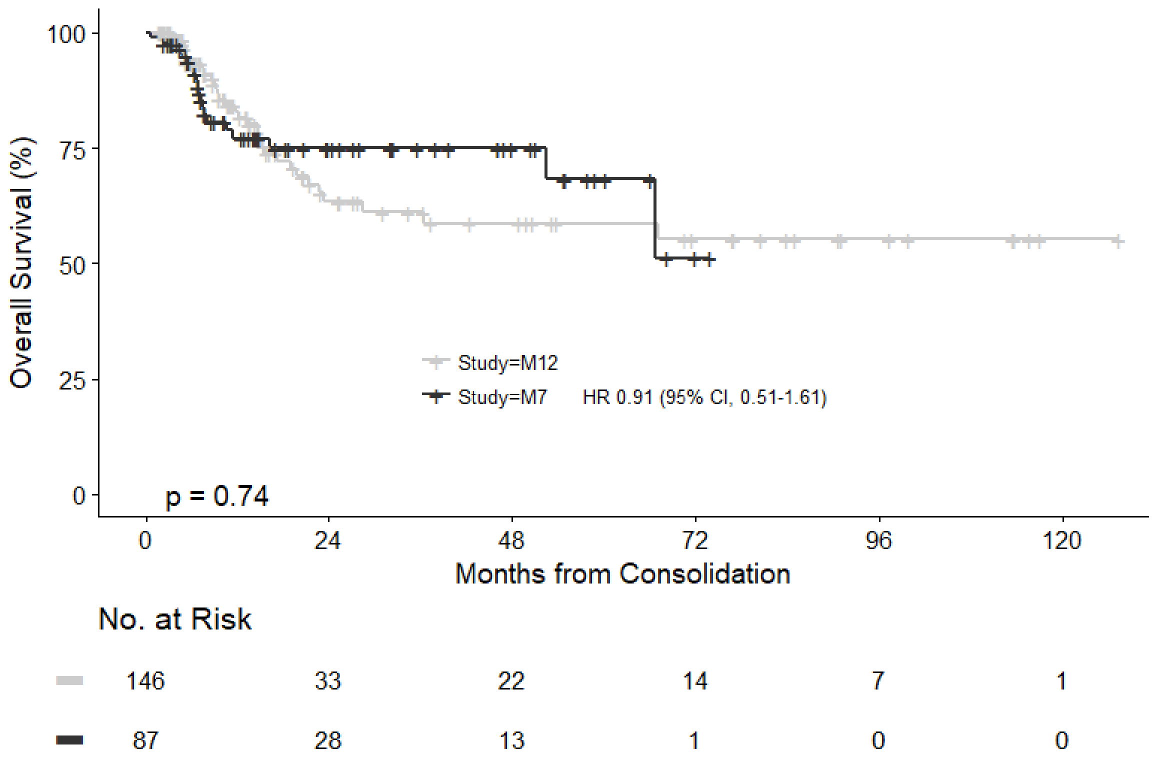

B

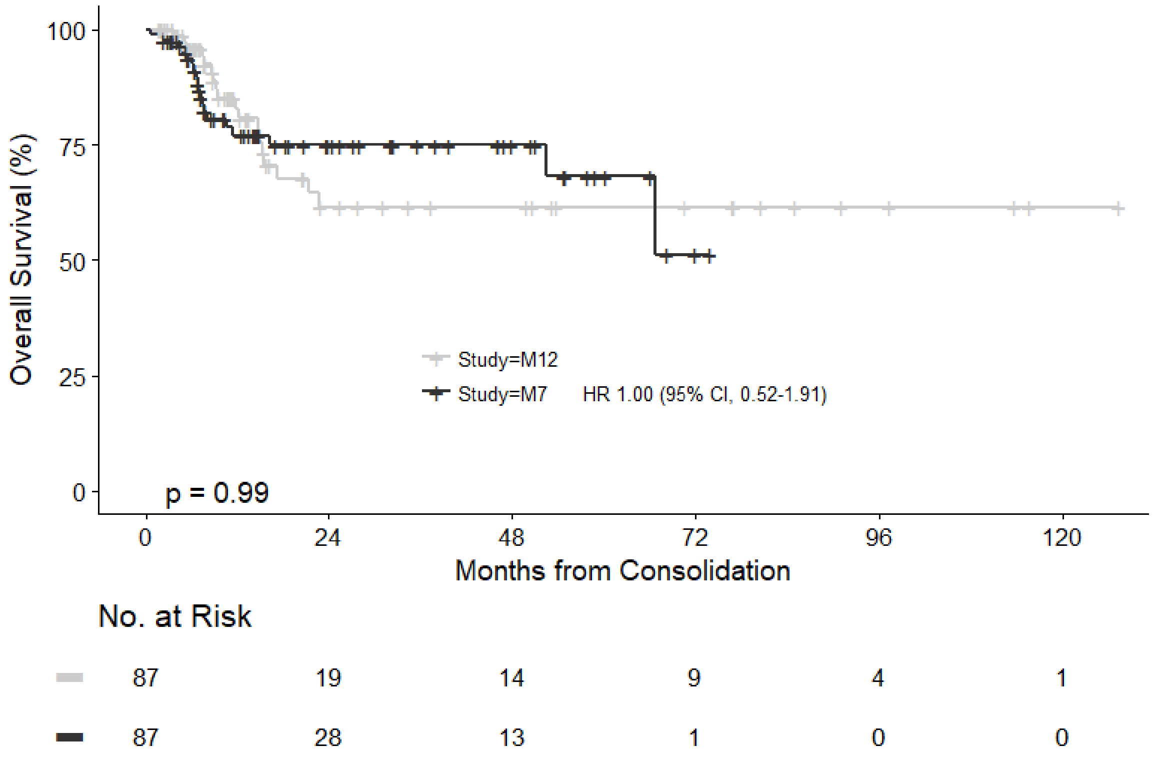

Figure S3. Cumulative incidence of relapse in patients receiving standard IcE consolidation in the AMLM7 and AMLM12 study cohorts, **(A)** ignoring SCT status (both autologous and allogeneic) and **(B)** treating SCT as competing risk.

A

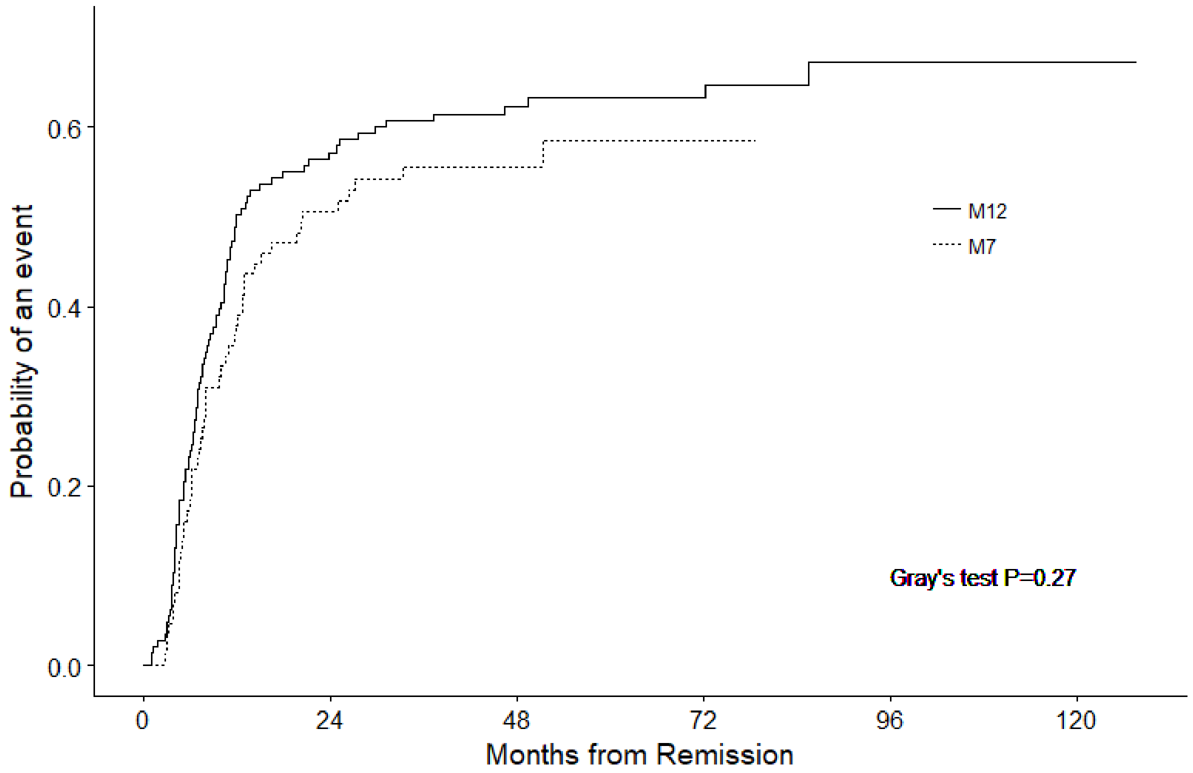

B

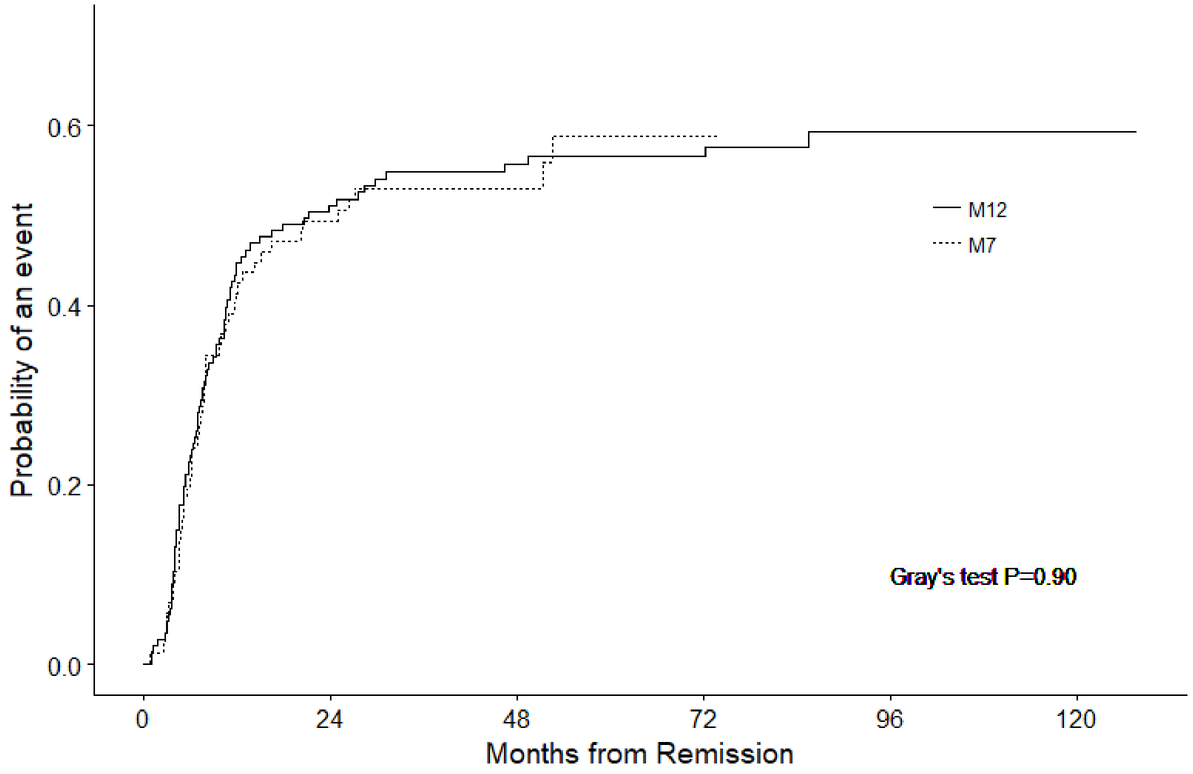

Figure S4. Overall survival in the AMLM7 study cohort receiving standard IcE consolidation chemotherapy, restricted to patients with >90 days of remission duration, stratified by **(A)** allogeneic SCT and **(B)** autologous SCT in first remission. Pairwise comparisons using log-rank test, with p-values adjusted by Benamini & Hochberg (BH) method.

A

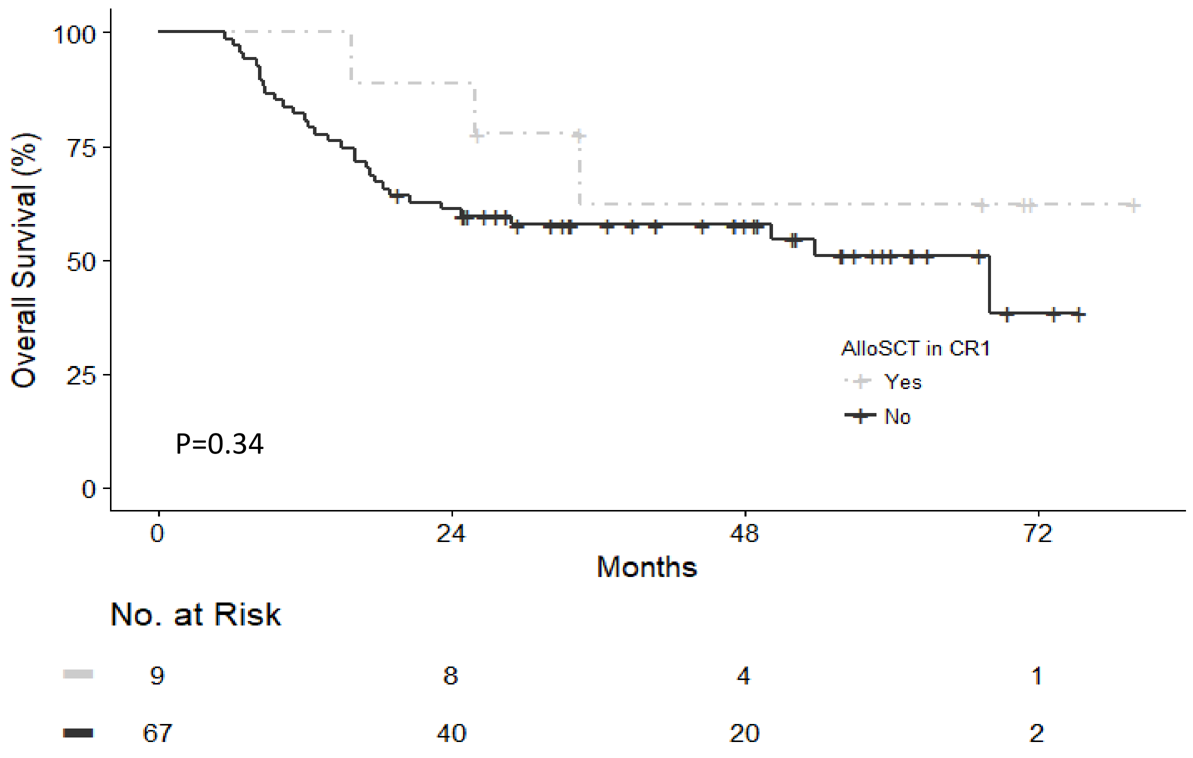

B

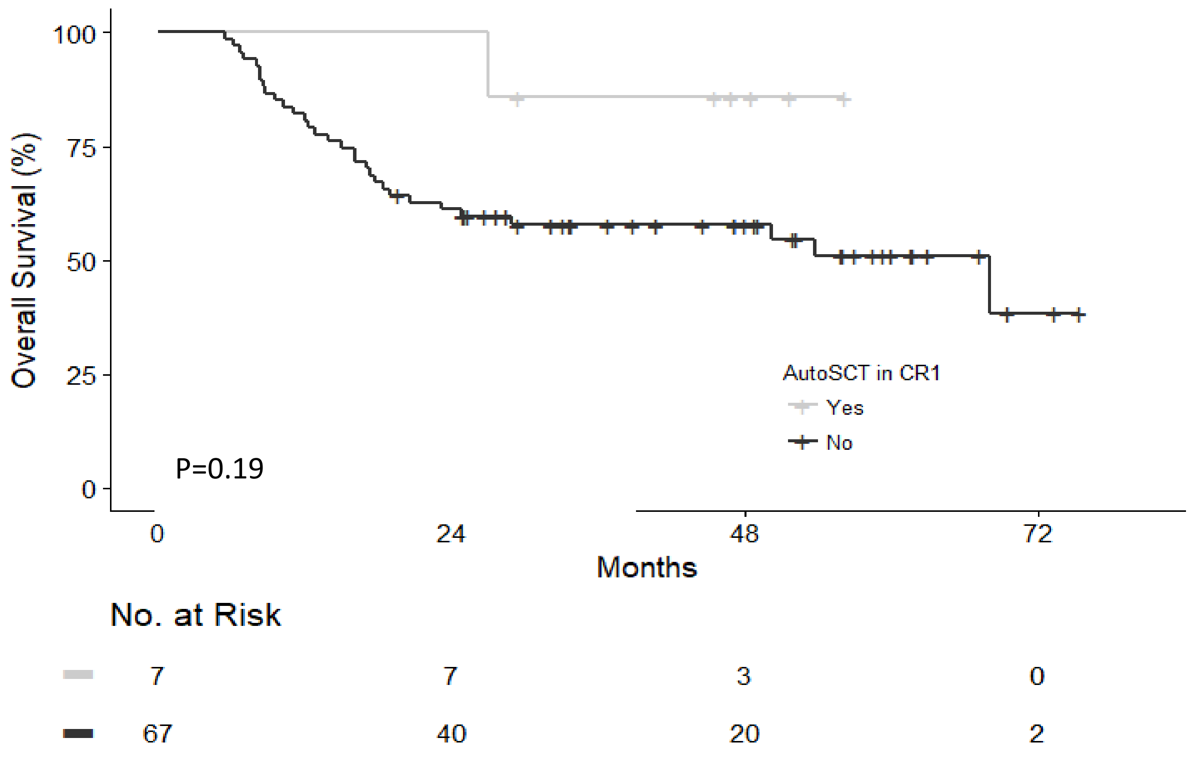

Figure S5. Overall survival after initial relapse of acute myeloid leukaemia, **(A)** in the entire AMLM7 and AMLM12 study cohorts and **(B)** following subsequent allogeneic stem cell transplantation in patients surviving >90 days from relapse.

A

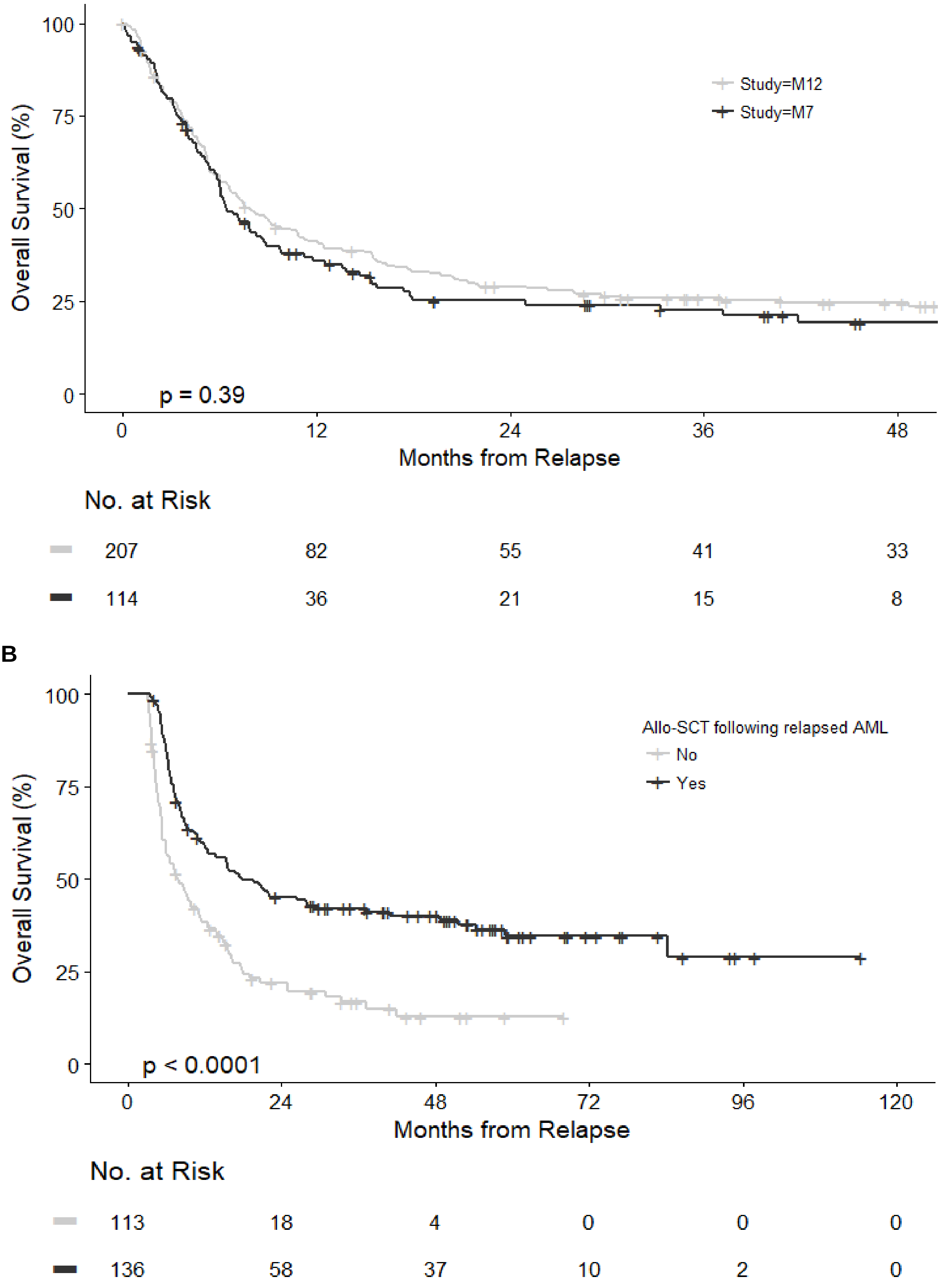

Figure S6. Overall survival according to recruiting volume in the **(A)** AMLM12 and **(B)** AMLM7 study cohorts.

A

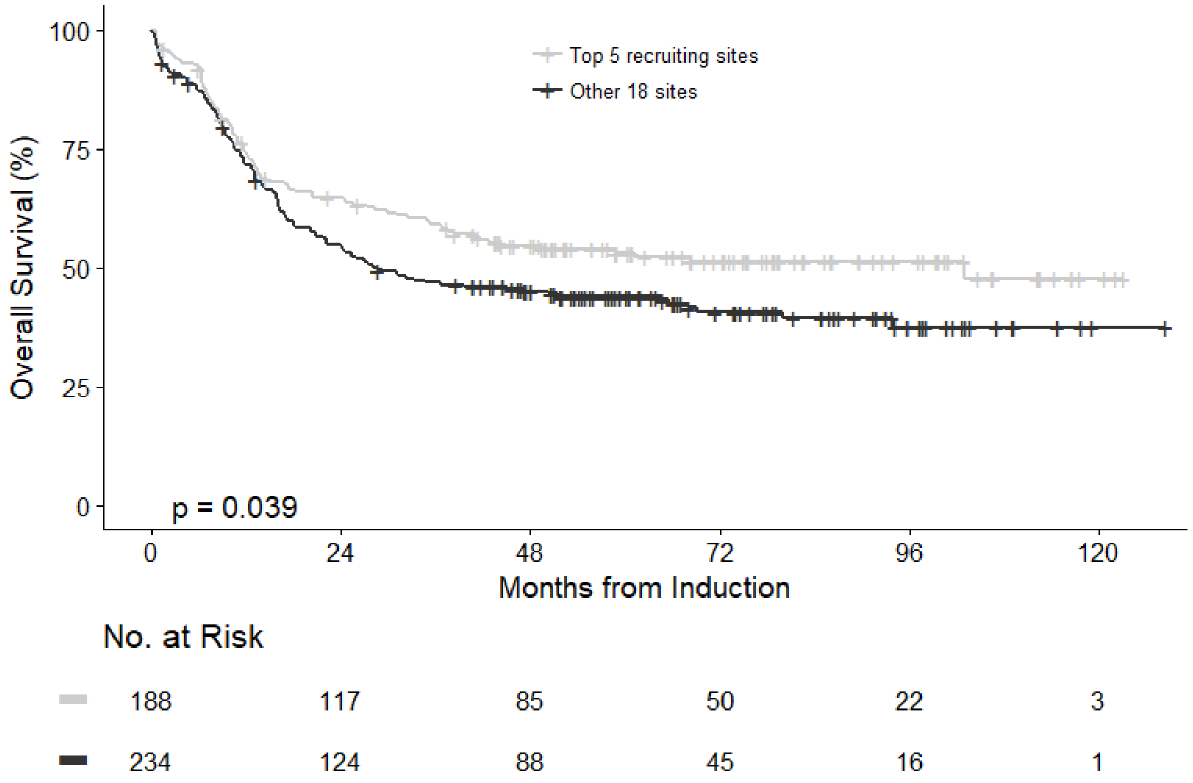

B

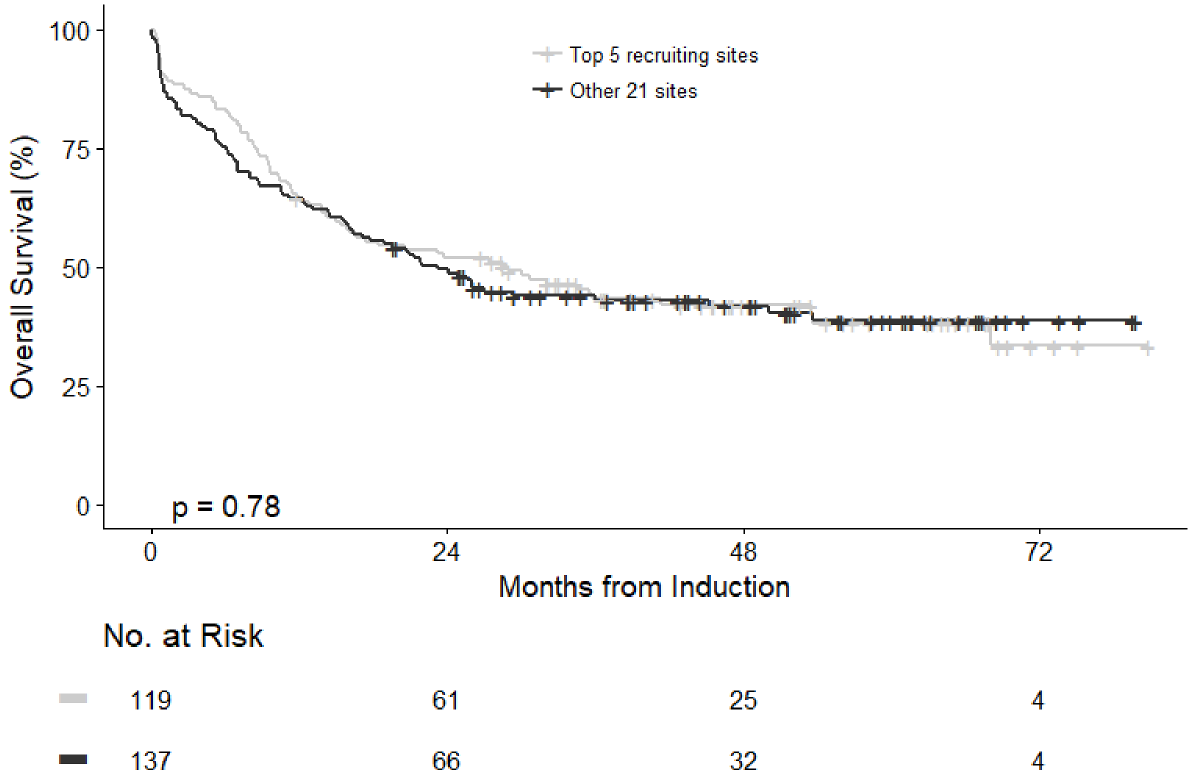

Supplement: Supplementary file 1 — Supplementary Material [file 41408_2018_121_MOESM1_ESM.pdf]
